# Analysis of motor control strategy for frontal and sagittal planes of circular tracking movements using visual feedback noise from velocity change and depth information

**DOI:** 10.1371/journal.pone.0241138

**Published:** 2020-11-11

**Authors:** Geonhui Lee, Woong Choi, Hanjin Jo, Wookhyun Park, Jaehyo Kim

**Affiliations:** 1 Department of Mechanical and Control Engineering, Handong Global University, Pohang, Republic of Korea; 2 Department of Information and Computer Engineering, National Institute of Technology, Gunma College, Maebashi, Japan; Washington University in Saint Louis School of Medicine, UNITED STATES

## Abstract

We aim to investigate a control strategy for the circular tracking movement in a three-dimensional (3D) space based on the accuracy of the visual information. After setting the circular orbits for the frontal and sagittal planes in the 3D virtual space, the subjects track a target moving at a constant velocity. The analysis is applied to two parameters of the polar coordinates, namely, *ΔR* (the difference in the distance from the center of a circular orbit) and *Δω* (the difference in the angular velocity). The movement in the sagittal plane provides different depth information depending on the position of the target in orbit, unlike the task of the frontal plane. Therefore, the circular orbit is divided into four quadrants for a statistical analysis of *ΔR*. In the sagittal plane, the error was two to three times larger in quadrants 1 and 4 than in quadrants 2 and 3 close to the subject. Here, *Δω* is estimated using a frequency analysis; the lower the accuracy of the visual information, the greater the periodicity. When comparing two different planes, the periodicity in the sagittal plane was approximately 1.7 to 2 times larger than that of the frontal plane. In addition, the average angular velocity of the target and tracer was within 0.6% during a single cycle. We found that if the amount of visual information is reduced, an optimal feedback control strategy can be used to reduce the positional error within a specific area.

## Introduction

Imitation movement based on visual information is important in motor learning tasks, such as daily movements, sports, and working with tools [1–5], and there have been various studies on human movements utilizing visual information guides [6–14]. Most of the research on tracking motion using visual guidance has been performed on a one-dimensional (1D) linear trajectory [12, 13] and two-dimensional (2D) circular trajectory [6–11, 14]. During these experiments, visual feedback regarding the positions and velocities of a target and a tracer was provided [6–14].

However, human motion is performed in a three-dimensional (3D) space, and includes movement in the sagittal plane, unlike tracking movements of 1D or 2D trajectories on the frontal plane. Depth information is required in order to determine the position of the target on the sagittal plane [15]. When performing a tracking movement that includes depth information, visual feedback on the position of the target is not sufficient [15–18]. Most of the previous studies analyzed the tracking motion from the frontal plane, and accordingly, the tracking motion analysis reflecting depth information was insufficient [6–14].

With the recent development of virtual reality (VR) technology, it was possible to implement various human movement experiments in a 3D virtual space. Some previous studies analyzed circular tracking motion in a 3D space, including depth information, using these VR devices [16–19]. In these studies, the tracking movement with constant velocity was conducted on the frontal and sagittal planes. One study analyzed positional errors in Cartesian coordinates [16]. Another study analyzed the three parameters *ΔR* (the difference in the distance from the fixed pole), *Δθ* (the difference in the position angle), and *Δω* (the difference in the angular velocity) in terms of the polar coordinates [17]. It was reported that the positional error is influenced by the depth information, and the phase error(*Δθ*) is proportional to the target velocity.

In circular tracking movement, there is no difference in depth information between each target position on the frontal plane. However, in the case of the sagittal plane, there is a difference in depth information depending on each position of the target, which causes a difference in the visual information where the target exists in the orbit. In the previous studies using VR, the average value of the parameters measured were analyzed without considering the difference in depth information according to the position of the target [16, 17]. For this reason, the previous studies were insufficient for analyzing the difference in visual feedback information [16, 17].

In this study, we analyzed the difference according to the depth information of a circular orbit divided into four quadrants in a circular tracking movement. The tracking movement along with the circular orbit has a constant periodicity on the frontal and sagittal planes. On the sagittal plane, the change in depth information varies with a constant periodicity. The experiment was conducted under four different velocity conditions in order to compare the difference according to the target velocity. The *ΔR* parameter was statistically analyzed in each quadrant. In addition, to confirm the periodicity, a frequency analysis was conducted on the fast Fourier transform (FFT) of the *Δω* parameter. We also investigated the visuo-motor control strategy according to the accuracy of the visual information from the viewpoint of the optimal feedback control (OFC).

## Materials and methods

### Subject

The subjects of the experiment were 26 adult men and women with an average age of 24.88 ± 3.18 years (Table 1). All subjects had normal or corrected-to-normal vision. None of the subjects had previously participated in a similar study. Four of the subjects were left-handed, and 22 subjects were right-handed (Table 1). All subjects received sufficient explanation regarding the experimental procedure and gave their written informed consent prior to the experiment. The protocol was approved by the ethics committee of the National Institute of Technology, Gunma College.

**Table 1:** Subject information: Age, sex, and dominant hand.

Subject no.	Age	Sex	Dominant Hand
1	25	M	Right
2	24	M	Right
3	24	M	Right
4	22	F	Right
5	22	F	Right
6	24	M	Left
7	26	M	Right
8	22	M	Right
9	22	F	Right
10	23	F	Left
11	23	F	Right
12	22	F	Right
13	35	M	Right
14	26	M	Right
15	25	M	Right
16	23	M	Right
17	26	M	Right
18	26	M	Right
19	24	M	Right
20	24	M	Right
21	23	M	Right
22	23	M	Left
23	25	M	Left
24	25	M	Right
25	33	M	Right
26	30	M	Right

### Experiment setup

**Fig 1:**
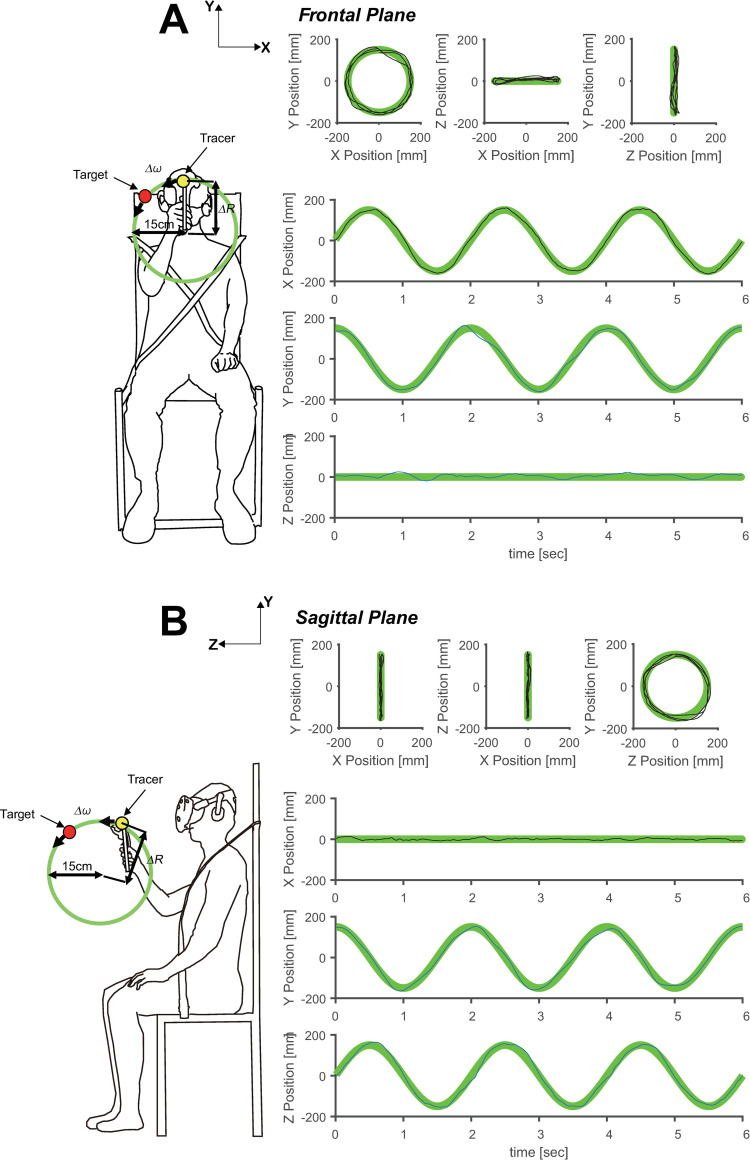
Experimental procedure. (A) Typical example of experimental setup and x-, y-, and z-axis data in the frontal plane. (B) Typical example of experimental setup and x-, y-, and z-axis data in the sagittal plane. The diagrams to the right of (A) and (B) show the plane position, subject’s rotational direction along the target, and the radius of the trajectory. The movement of the target was only shown to the subject during the experiment, and the pathway was invisible. The green line indicates the target’s movement, and the black solid line shows the tracking data of the subject.

We implemented a 3D VR environment using the Unity 3D program. An HTC VIVE Headset and a handheld controller were used as the interfaces [16]. The subjects were asked to conduct a circular tracking movement with visual guidance in a VR space, and used a tracer to track a target (Fig 1), namely, a red ball with a radius of 1.5 cm. The tracer was a yellow ball with a radius of 1 cm and a 20-cm-long stick. The position of the tracer was synchronized using the handheld controller, as shown in Fig 1. The upper limbs of the subjects were not displayed in the VR space. Therefore, the subjects received visual feedback of their hand position and direction through the tracer. Circular orbits were presented on the frontal and sagittal planes with a 15-cm radius. The moving pathway of the target was invisible to the subjects. In previous studies about tracking movement, the target velocities were set to 0.05~1.8Hz (Table 2). Accordingly, these studies inspired us to select target speeds of four target speeds: *V1* = 0.125, *V2* = 0.25, *V3* = 0.5, and *V4* = 0.75 Hz.

**Table 2:** Target velocities in tracking movement studies.

	Previous studies	target speed [Hz] (target path)
**1**	Miall et al. (*Behav Brain Res*.) 1986 [20]	0.05–1.0 Hz (pseudorandom waveforms on 1D space)
	Miall et al. (*Behav Brain Res*.) 1988 [21]	0.1, 0.2, 0.3, 0.4, 0.45, 0.5, 0.55, 0.6 Hz (sinusoids waveforms on 1D space)
	Miall et al. (*J Mot Behav*.) 1993 [22]	0.04, 0.06, 0.08, 0.167 Hz (pseudorandom and sinusoids waveforms on 1D space)
**2**	Ishida and Sawada (*Physical review letters*) 2004 [23]	0.1, 0.5, 0.8, 1.3, 1.8 Hz (sinusoids waveforms on 1D space)
**3**	Inoue and Sakaguchi et al. (*Neural Networks*) 2015 [24]	0.073, 0.117, 0.205, 0.278, 0.3 Hz (pseudorandom and sinusoids waveforms on 1D space)
**4**	Hayashi et al. (*Artif Life Robotics*) 2009 [25]	0.1, 0.2, 0.3, 0.4, 0.5, 0.6, 0.7 Hz (circular trajectory on 2D space)
**5**	Kim et al. (*Advanced Robotics*) 2017 [10]	0.05, 0.1, 0.2 Hz (circular trajectory on 2D space)

In this study, the target was moved clockwise in the circular orbit when the right-handed subjects were performing the experiment, and counterclockwise in the circular orbit when the left-handed subjects were performing the experiment. The reason for using the counterclockwise direction with the left-handed subjects is to allow them to perform the same movement based on the human internal coordinate system.

The subjects wore a VIVE headset and held a controller with their dominant hand while sitting on a chair. The distance between the eye and the screen of the VIVE headset was adjusted according to the criteria of each subject. The upper body of the subject was fastened using a belt to limit shoulder movement.

We applied a method for calibrating the initial position of the target optimized for the subject’s arm length and height to minimize the effect of the different anthropometric parameters on the experimental results. The initial position of the target was calibrated before the experiments because the height and arm length of each subject were different.

**Fig 2:**
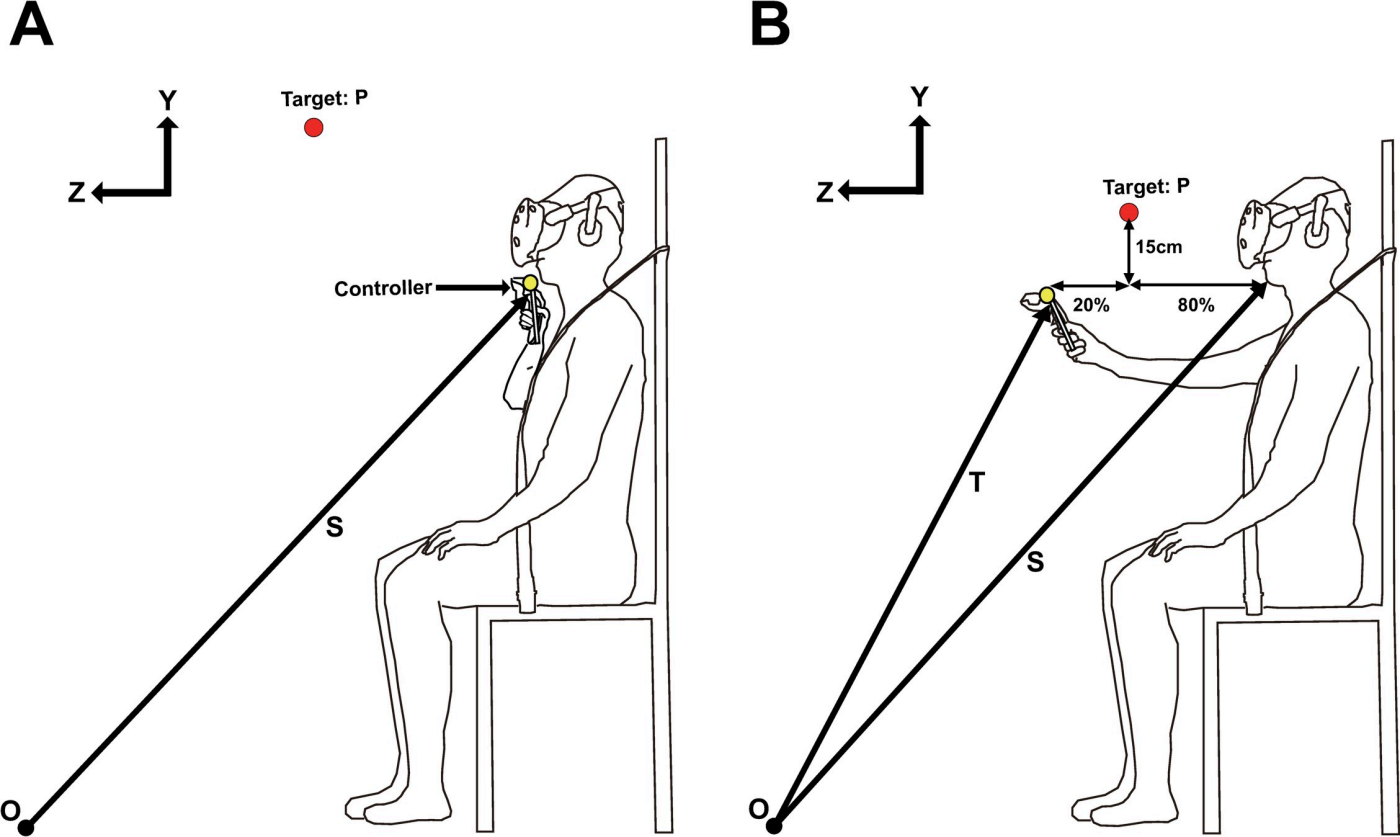
Calibration procedure. (A) Initialization of target x-axis position (B) Initialization of target y-axis, z-axis position. Process (A) is performed first, and then process (B) is performed to set the initial position of the x, y, and z axes of the target.

*P*(*p*_*x*_,*p*_*y*_,*p*_*z*_) is the coordinate of the target, and *p*_*x*_,*p*_*y*_,*p*_*z*_ indicates the coordinates for the x-axis [m], y-axis [m], and z-axis [m] of the target. The chin position of the subject was measured, and the initial *P*_*x*_ position was set based on this (Fig 2A). The system measured the stretched position of the subject’s arm length and initialized *P*_*y*_, *P*_*z*_ (Fig 2B). *P*_*x*_ was taken as the initial position on the same line as the chin. The initial *P*_*y*_ position was set to 15 cm above the center of the circle (Fig 2B). *P*_*z*_ was determined considering the case where the subject performed a tracking motion on the sagittal plane. Using the stated calibration process, feasible ranges of motion of individual subjects were determined in order to practice the tracking movements without the need to fully stretch the multi-joint upper limb. Moreover, a virtual trajectory of the target could be generated at an appropriate distance from the head-mounted display (HMD) to prevent any potential contact or penetration of the target on the subject’s body. Accordingly, the target was positioned 20% closer to the body from the position of the subject's stretched arm (Fig 2B). The two constants (15 cm, 20%) were set according to a preliminary test to ensure that the subject had a safe and comfortable working space.

Before starting the main experiment, the subject was familiarized with the VR space using a pre-experiment. The experiment was started by providing an auditory signal to the subject after a 3-s countdown. One trial consisted of five revolutions of the circle. All subjects continuously conducted five trials of the circular tracking movement at the same velocity, and were given a break for 5 min before the next five trials. Therefore, a total of 40 experimental trials were conducted (five trials with 4 velocities × 2 planes) for each subject. To avoid the subjects’ learning effects, the experiment was executed using a random counterbalance for both the planes and velocity of the target.

### Data analysis

The positional data based on the Cartesian coordinates from the tracer and the target were measured during each task. For the data analysis, the *x*, *y*, and *z* data were converted into the radial and angular velocities of the polar coordinates, and were designated as *R* and *ω*, respectively. In addition, the difference in each parameter was calculated to compare the differences in movements between the tracer and the target. The experiment was performed using five trials under each condition; however, the first and last trials were excluded from the analysis, because they were considered to be transient sections.

$$\Delta R(t)[mm]=|R_{tracer}(t)-R_{target}|$$ (1)

$$\Delta R_{Quadrant1}=\frac{\sum_{t=1}^{n/12}\left\{\Delta R(t)+\Delta R(t+\frac{n}{3})+\Delta R(t+\frac{2n}{3})\right\}}{n/4}$$ (2)

$$\Delta R_{Quadrant2}=\frac{\sum_{t=\frac{n}{12}+1}^{n/6}\left\{\Delta R(t)+\Delta R(t+\frac{n}{3})+\Delta R(t+\frac{2n}{3})\right\}}{n/4}$$ (3)

$$\Delta R_{Quadrant3}=\frac{\sum_{t=n/6+1}^{n/4}\left\{\Delta R(t)+\Delta R(t+\frac{n}{3})+\Delta R(t+\frac{2n}{3})\right\}}{n/4}$$ (4)

$$\Delta R_{Quadrant4}=\frac{\sum_{t=n/4+1}^{n/3}\left\{\Delta R(t)+\Delta R(t+\frac{n}{3})+\Delta R(t+\frac{2n}{3})\right\}}{n/4}$$ (5)

*ΔR* is defined as the difference in the absolute radial distance from the origin between the target and the tracer. This can be confirmed using Eq (1). The values of *ΔR* were classified into four quadrants, and were calculated as the average value for all subjects in the same quadrant for each trial. Eqs (2) to (5) represent the definitions of the average values of *ΔR* in each quadrant. The constant *n* represents the total time of the three trials at each target velocity. We calculated the mean (*M*) and standard deviation (*SD*) of the normalized *ΔR* for the 26 subjects.

$$\Delta \omega (t)\;[deg/s]=|\omega_{tracer}(t)-\omega_{target}(t)|$$ (6)

$$\omega_{average}(t)=\frac{\sum_{k=1}^{m}\omega_{k}(t)}{m}$$ (7)

In Eq (6), *Δω* is defined as the absolute difference in the angular velocity between the target and the tracer. This parameter was calculated as the mean value for 26 subjects, where *m* represents the total number of subjects.

In this study, we investigated the relationship between the velocity of the target and the quadrant position during 3D target tracking movements. Therefore, the data on *ΔR* were statistically analyzed using a two-way repeated analysis of variance (*ANOVA*) for the quadrants (with four levels, i.e., quadrant 1, Q1, quadrant 2, Q2, quadrant 3, Q3, and quadrant 4, Q4), and velocity (with four levels of *V1* = 0.125, *V2* = 0.25, *V3* = 0.5, and *V4* = 0.75 Hz) factors. Statistical analyses and data visualization were conducted using SPSS Statistics V26 (IBM) and MATLAB (MathWorks).

In this study, the subjects repeatedly conducted circular tracking movements. In particular, it is necessary to check the periodicity on the sagittal plane because the depth information of the target changes periodically. To do this, *Δω* was analyzed using the FFT, which is shown in Eq (8), where *n* represents the total time for three trials at each target velocity.

The FFT analysis of *Δω* was conducted after pre-processing with a low-pass filter (LPF). The cutoff frequency was determined to be 5 Hz, which can sufficiently measure the movement of the human body [26, 27].

$$f_{j}=\sum_{k=0}^{n-1}x_{k}e^{-\frac{2\pi i}{n}jk}\;j=0,\ldots ,n-1$$ (8)

## Results

**Fig 3:**
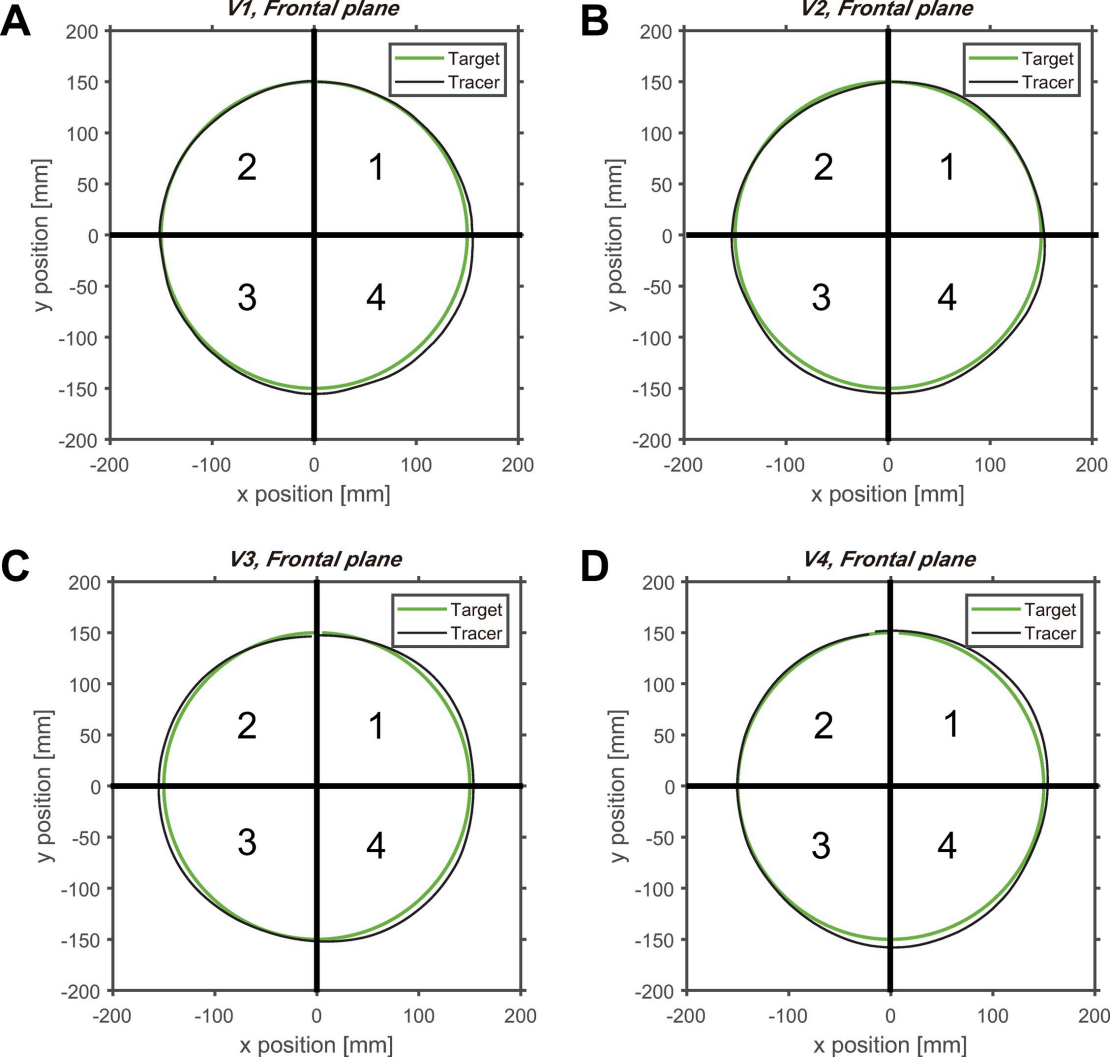
Average circular tracking movement in the frontal plane. (A) The movement trajectory of the target and controller at (A) V1 (0.125 Hz), (B) V2 (0.25 Hz), (C) V3 (0.5 Hz), and (D) V4 (0.75 Hz). The order of the moving target is the *Q1*, *Q4*, *Q3*, and *Q2*.

We compared the average trajectories of the target and tracer on each plane. Comparing the average orbit of the tracer with the orbit of the target on the frontal plane, we noticed that the orbits are mostly similar in all quadrants (Fig 3), with the difference being independent of the velocity of the target.

**Fig 4:**
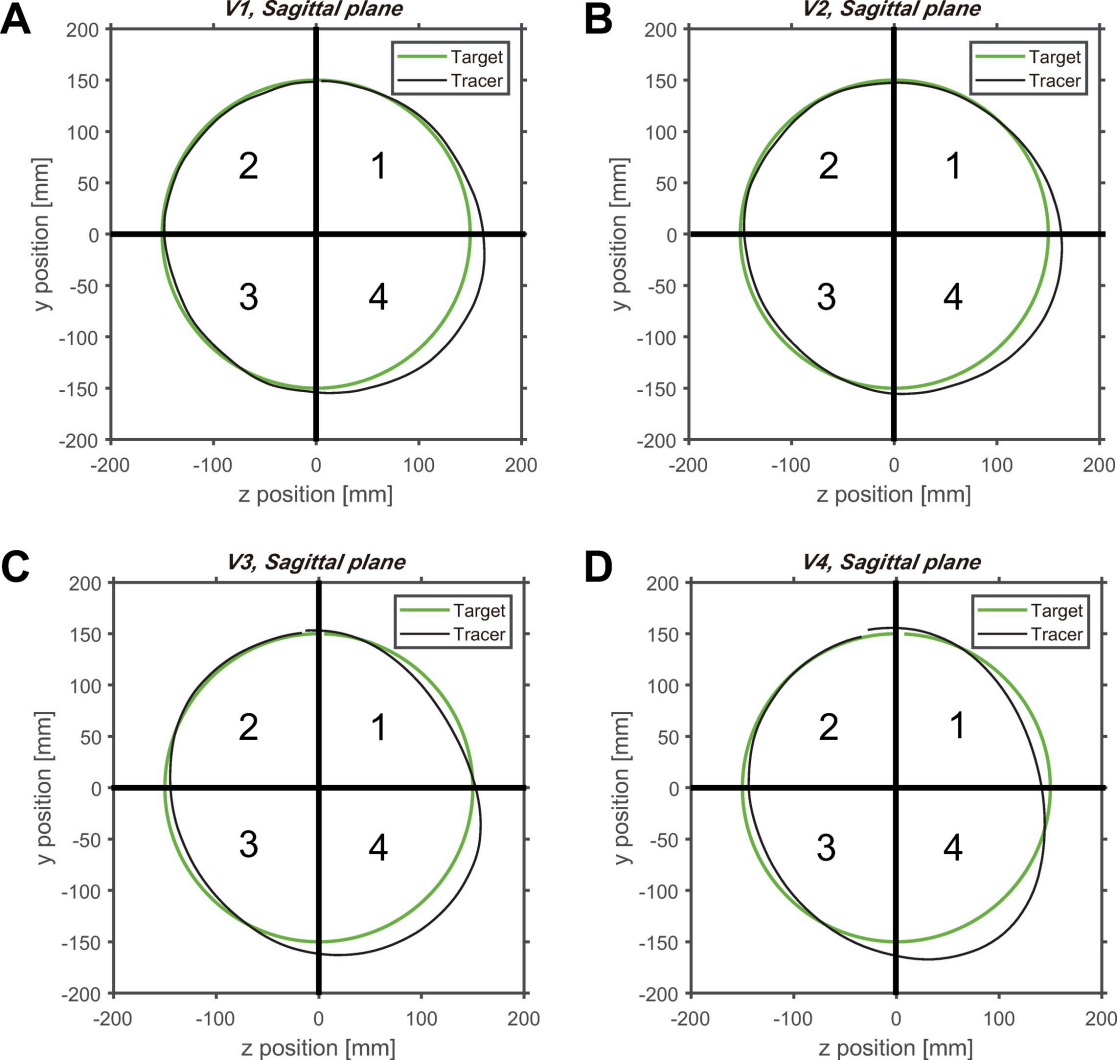
Average circular tracking movement in the sagittal plane. (A) The movement trajectory of the target and controller at (A) *V1* (0.125 Hz), (B) *V2* (0.25 Hz), (C) *V3* (0.5 Hz), and (D) *V4* (0.75 Hz). The order of the moving target is the *Q1*, *Q4*, *Q3*, and *Q2*.

When comparing the trajectories of the tracer and target on the sagittal plane, there is a large *ΔR* in the fourth quadrant, regardless of the velocity (Fig 4). This indicates that errors in the tracer and target trajectory varied depending on the quadrant in which the tracer was located.

### Analysis of *ΔR* between frontal and sagittal planes

**Fig 5:**
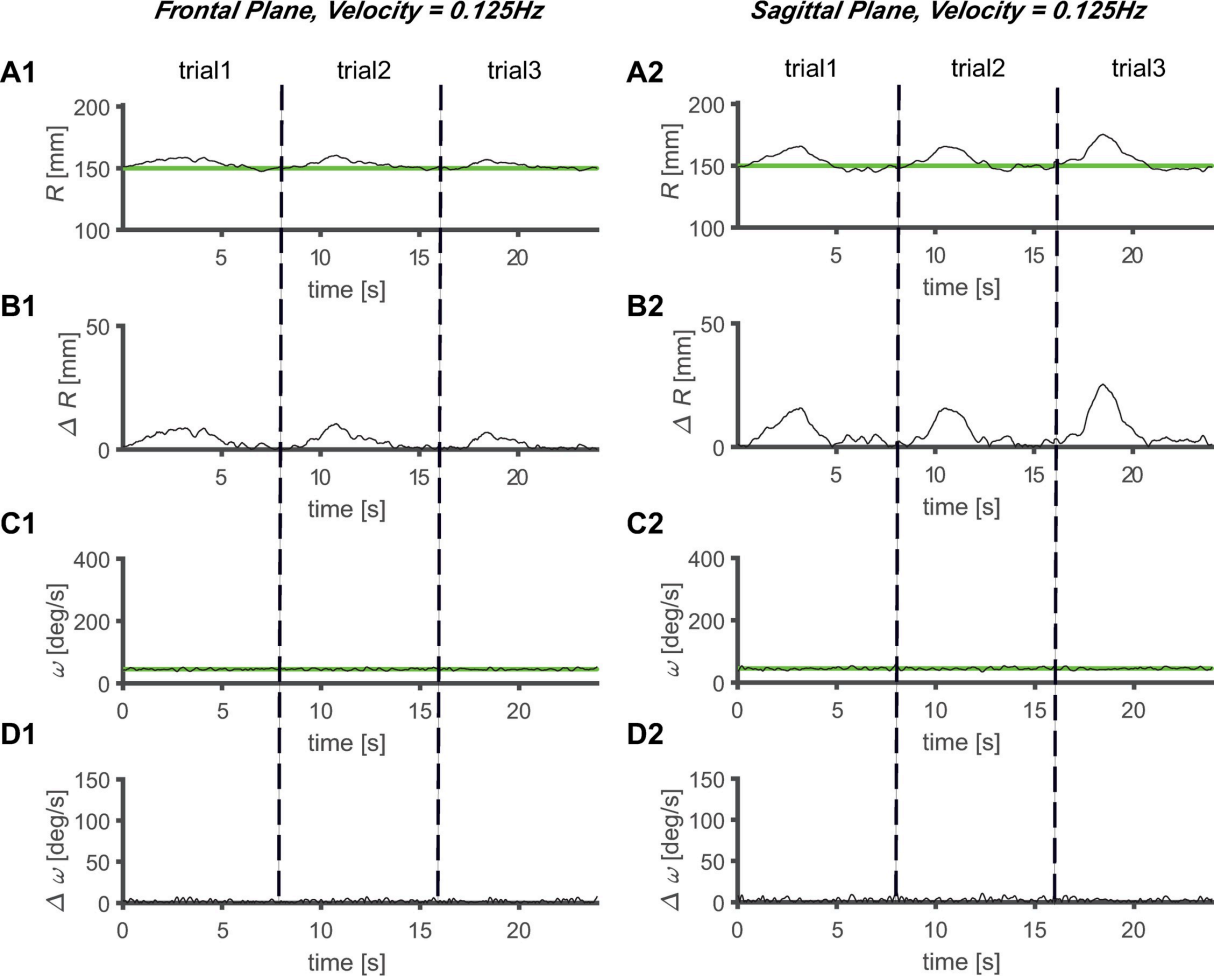
Average circular tracking movement at 0.125 Hz. The R value for three trials on the (A1) frontal plane (A1) and (A2) sagittal plane. Absolute value of ΔR on the (B1) frontal plane (B1) and (B2) sagittal plane, ω value for three trials on the (C1) frontal plane (C1) and (C2) sagittal plane, and absolute value of Δω on the (D1) frontal plane (D1) and (D2) sagittal plane.

**Fig 6:**
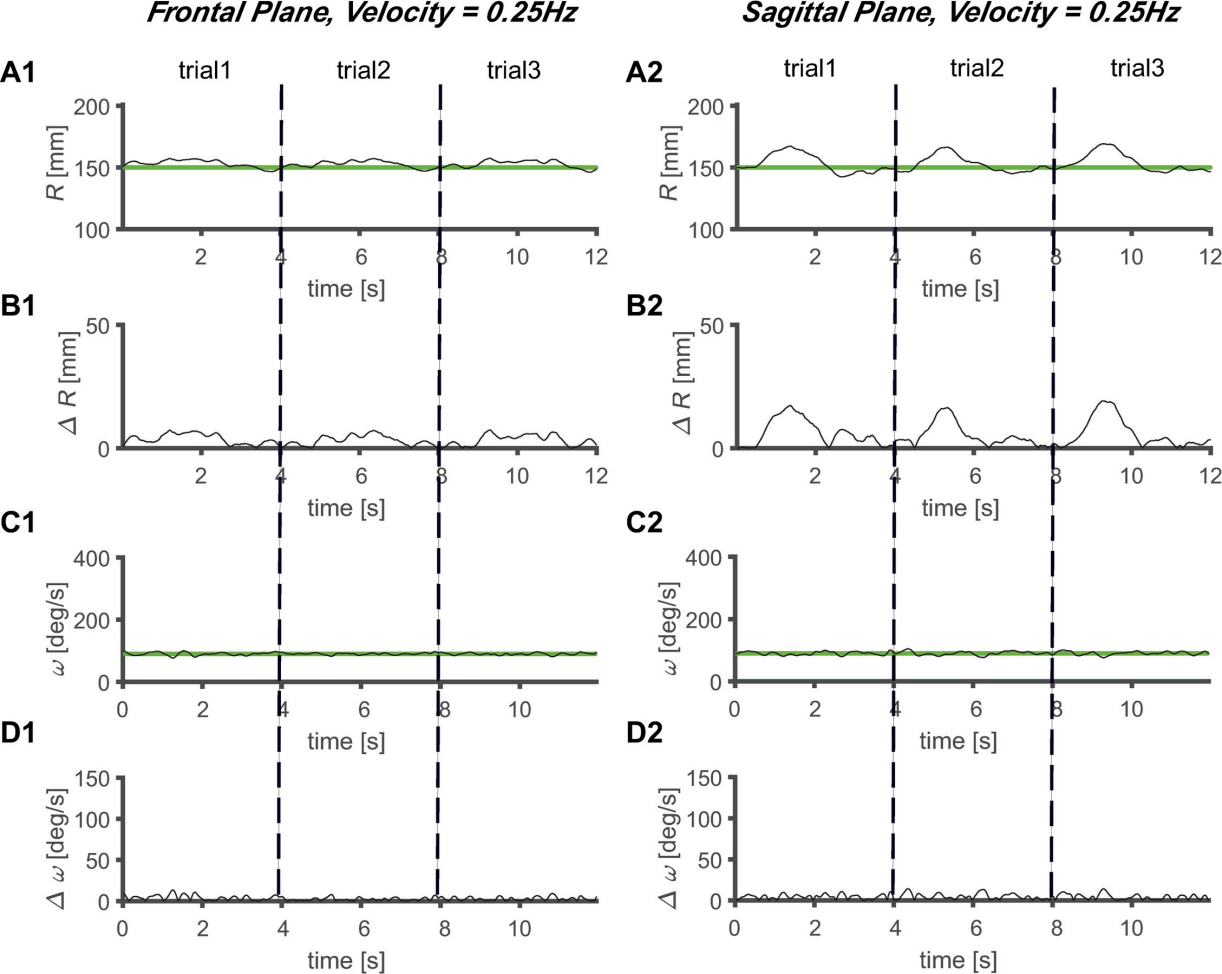
Average circular tracking movement at 0.25 Hz. *R* value for three trials on the (A1) frontal plane (A1) and (A2) sagittal plane. Absolute value of *ΔR* on the (B1) frontal plane (B1) and (B2) sagittal plane. *ω* value for three trials on the (C1) frontal plane (C1) and (C2) sagittal plane. Absolute value of *Δω* on the (D1) frontal plane (D1) and (D2) sagittal plane.

**Fig 7:**
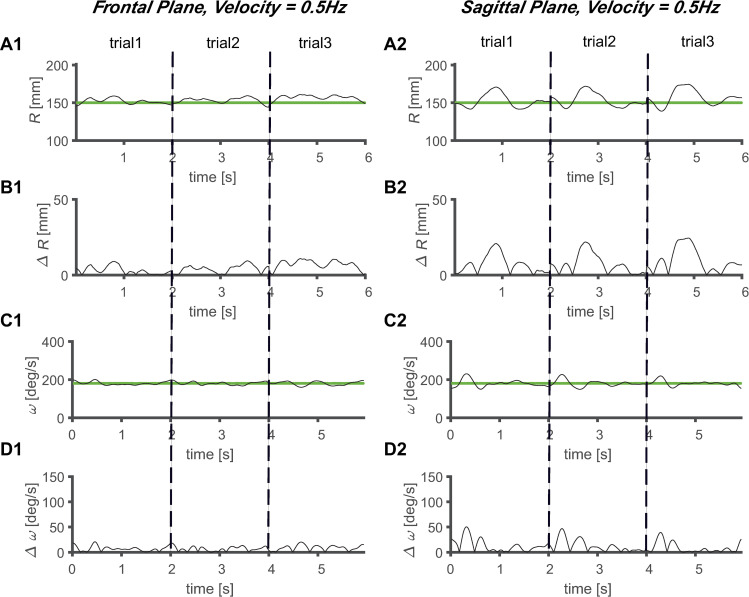
Average circular tracking movement at 0.5 Hz. *R* value for three trials on the (A1) frontal plane (A1) and (A2) sagittal plane. Absolute value of *ΔR* on the (B1) frontal plane (B1) and (B2) sagittal plane. *ω* value for three trials on the (C1) frontal plane (C1) and (C2) sagittal plane. Absolute value of *Δω* on the (D1) frontal plane (D1) and (D2) sagittal plane.

**Fig 8:**
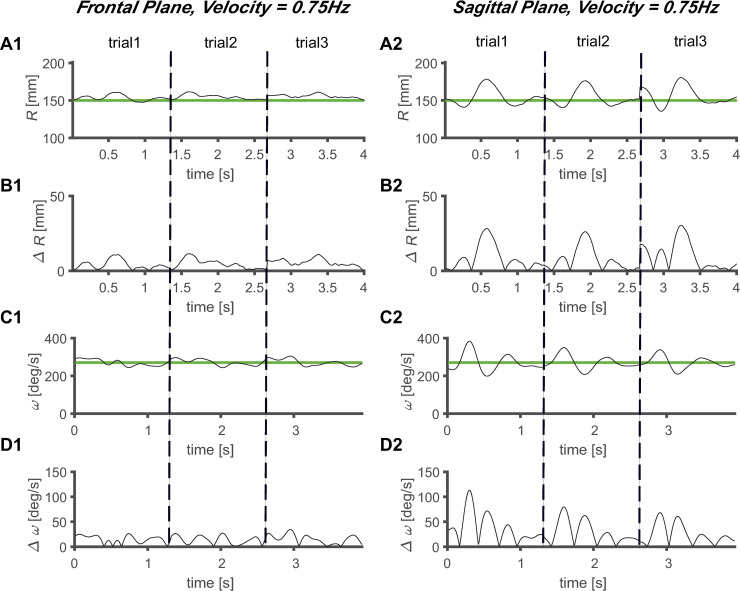
Average circular tracking movement at 0.75 Hz. *R* value for three trials on the (A1) frontal plane (A1) and (A2) sagittal plane. Absolute value of *ΔR* on the (B1) frontal plane (B1) and (B2) sagittal plane. *ω* value for three trials on the (C1) frontal plane (C1) and (C2) sagittal plane. Absolute value of *Δω* on the (D1) frontal plane (D1) and (D2) sagittal plane.

The magnitude of *ΔR* increased as the velocity of the target increased, regardless of the plane of the task (Figs 5–8). Comparing the frontal and sagittal planes at the same velocity, a larger *ΔR* was shown on the sagittal plane (Figs 5–8). In addition, the *ΔR* value of the target and the tracer on the sagittal plane was noticeable at certain points, indicating that the size of *ΔR* varies depending on the trajectory position of the tracer and target.

On the frontal plane, there was a significant difference between the velocity of the target (*F* (1.776, 44.399) = 48.675, *p* = 0, partial *η*^2^ = 0.661, item A in S1 Table) and the quadrant of the orbit (*F* (2.166, 54.143) = 9.658, *p* = 0, partial *η*^2^ = 0.279, item A in S1 Table). On the sagittal plane, there were significant differences between the velocity of the target (*F* (3, 75) = 34.941, *p* = 0, partial *η*^2^ = 0.583, item A in S2 Table) and quadrants of the orbit (*F* (2.092, 52.288) = 56.260, *p* = 0, partial *η*^2^ = 0.692, item A in S2 Table). These results indicate that the quadrant and velocity factors affect the value of *ΔR* during the circular tracking movement. In particular, the significant difference in each quadrant indicates that there is a difference in the control of the *ΔR* value according to the track position on the same plane.

**Fig 9:**
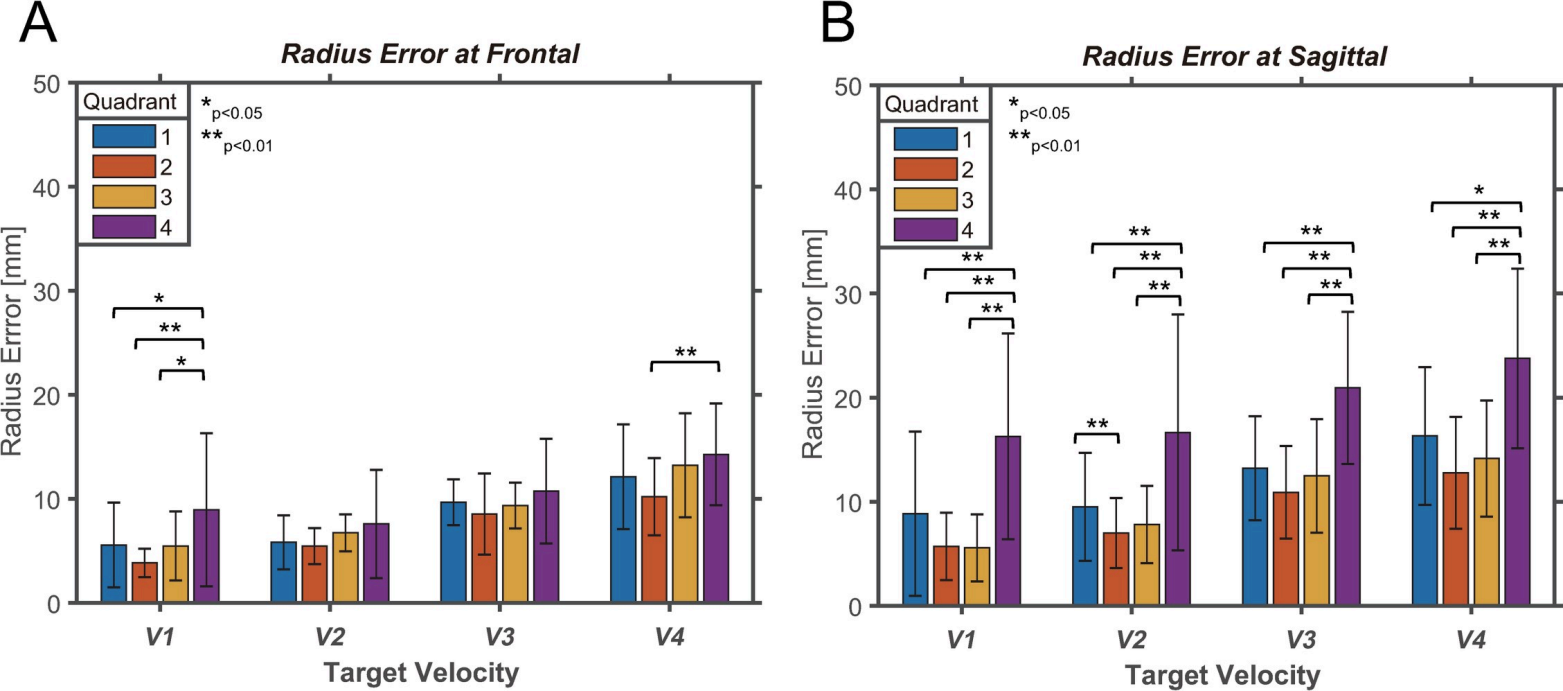
Evaluation of *ΔR* in circular tracking movement. (A) Pairwise comparisons are shown for *ΔR* between four quadrants on the frontal plane. (B) Pairwise comparisons are indicated for *ΔR* between four quadrants on the sagittal plane.

Next, a pairwise comparison was conducted to analyze the control performance between quadrants. As shown in Fig 9A, there was a significant difference in *ΔR* in the quadrant of the frontal plane at *V1* and *V4*. There was no significant difference between quadrants at *V2* and *V3*, when comparing the quadrants for each velocity. At *V1*, there was a significant difference between quadrant 4 and the other quadrants. For a velocity of 4, there was a significant difference between quadrants 4 and 2 (items B–E in S1 Table). On the frontal plane, the difference between the fourth quadrant with the largest *ΔR* and the second quadrant with the smallest *ΔR* averaged approximately 3.38 mm (Table 3). However, on the sagittal plane, the *ΔR* value between the second and fourth quadrants was 10.32 mm on average (Table 3), which was approximately three times higher than on the frontal plane. This implies that there is some difference in *ΔR* between the four quadrants on the frontal plane; however, it is not noticeable when compared to that on the sagittal plane.

As shown in Fig 9B, a significant difference in *ΔR* in the quadrant is shown for *V1*, *V2*, *V3*, and *V4* on the sagittal plane. There were significant differences between the fourth quadrant and the other quadrants, regardless of the velocity, when comparing the quadrants at each velocity. In addition, in the first and fourth quadrants, the errors in the radius tend to be larger than those in the second and third quadrants. According to the statistical analysis, the *ΔR* value in the fourth quadrant was significantly different from that in the other quadrants, and as aforementioned, the *ΔR* value of the sagittal plane was approximately three times higher than that of the frontal plane. We found that the difference in *ΔR* between the quadrants on the sagittal plane was more noticeable than that of the frontal plane, and the *ΔR* value tended to increase as the distance between the subject and the target increased.

**Table 3:** Average *ΔR* values between the target and the tracer, and standard deviation of *ΔR*.

Plane	Quadrant	V1	V2	V3	V4
**Frontal**	**Q1**	5.57 ± 0.80	5.84 ± 0.51	9.68 ± 0.43	12.13 ± 0.98
	**Q2**	3.85 ± 0.27	5.46 ± 0.34	8.55 ± 0.77	10.21 ± 0.73
	**Q3**	5.48 ± 0.65	6.75 ± 0.35	9.37 ± 0.43	13.24 ± 0.98
	**Q4**	8.95 ± 1.44	7.60 ± 1.02	10.76 ± 0.99	14.28 ± 0.96
	**Q4 –Q2**	**5.10**	**2.14**	**2.21**	**4.07**
**Sagittal**	**Q1**	8.85 ± 1.55	9.50 ± 1.02	13.21 ± 0.98	16.31 ± 1.30
	**Q2**	5.70 ± 0.63	6.98 ± 0.66	10.90 ± 0.87	12.77 ± 1.05
	**Q3**	5.56 ± 0.63	7.80 ± 0.73	12.48 ± 1.07	14.14 ± 1.09
	**Q4**	16.28 ± 1.94	16.65 ± 2.22	20.93 ± 1.44	23.77 ± 1.69
	**Q4 –Q2**	**10.58**	**9.67**	**10.03**	**11**

### Analysis of *Δω* between frontal and sagittal planes

The values of *Δω* increased as the velocity increased, regardless of the plane in which the task was performed (Figs 5–8). At the same velocity, the values of *Δω* were larger on the sagittal plane than on the frontal plane (Figs 5–8). During the three trials, the *ω* values of the tracer were maintained at substantially constant values at *V1* and *V2*, and at *V3* and *V4*, it was observed that the *ω* values of the tracer changed to a sinusoidal shape over time, and periodicity was observed in the variation (Figs 5–8). This shows that the *ω* values change with periodicity when the target moves fast (*V* ≥ 0.5 Hz).

**Fig 10:**
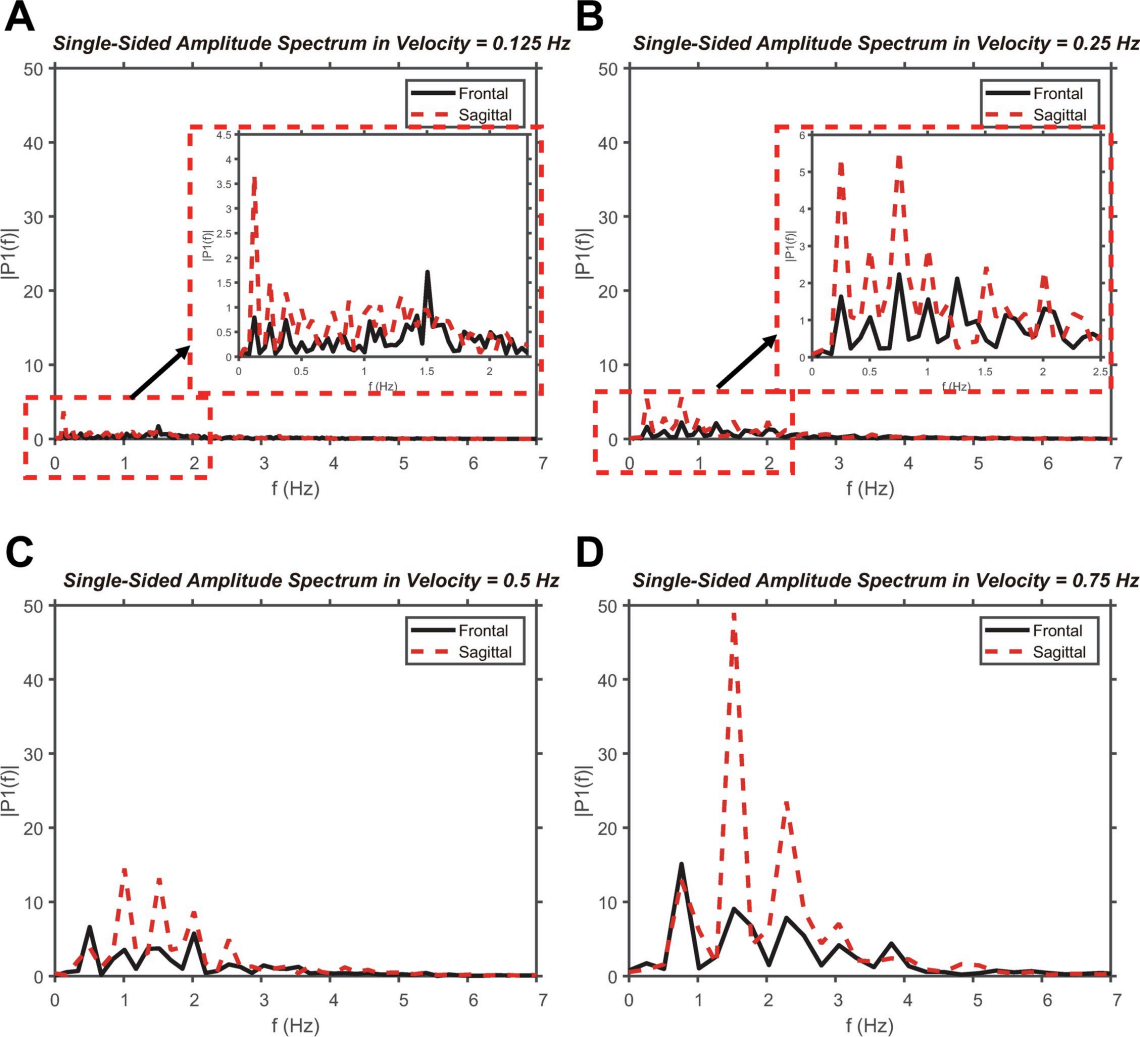
Frequency analysis of ω. Analysis of the *ω* values of the frontal and sagittal planes within the frequency domain at (A) *V1*, (B) *V2*, (C) *V3*, and (D) *V4*.

Fig 10 shows the *ω* values for each velocity on the frontal and sagittal planes in the frequency domain. At *V1* (Fig 10A) and *V2* (Fig 10B), the amplitude spectrum is between 0 and 2.5 Hz, and the area of the amplitude spectrum (1.37 Hz∙deg/s (*V1*) and 2.74 Hz∙deg/s (*V2*) on the frontal plane, and 2.13 Hz∙deg/s (*V1*) and 4.53 Hz∙deg/s (*V2*) on the sagittal plane) is smaller than the amplitude spectrum area (8.15 Hz∙deg/s (*V3*) and 22.84 Hz∙deg/s (*V4*) on the frontal plane, and 14.03 Hz∙deg/s (*V3*) and 39.97 Hz∙deg/s (*V2*) on the sagittal plane) at *V3* and *V4*. At *V3* (Fig 10C) and *V4* (Fig 10D), the amplitude spectrum appears between 0 and 5 Hz, which is relatively large compared to the values at *V1* and *V2*. In addition, it can be seen that the amplitude spectrum area of the sagittal plane is larger than the amplitude spectrum area of the frontal plane regardless of the velocity. We found that the periodicity features were stronger on the sagittal plane than on the frontal plane. We propose that depth information affects the periodicity of *ω*.

Table 4 compares the average *ω* values of the target and tracer during each trial on the frontal and sagittal planes. Regardless of the velocity and plane, the average *ω* values of the target and multiple tracers were almost the same, and the error was within 0.5%. The maximum standard deviation was 3.2 deg/s, and there was no significant difference in the average *ω* among the subjects. This means that the time of one trial is almost the same for both the tracer and target. Therefore, the period of one trial is a more important control parameter than the instantaneous *ω* value of the tracer in orbit during a repeated circular tracking movement.

**Table 4:** Average *ω* value of target and tracer, and standard deviation of tracer *ω* value.

Plane	Frontal				Sagittal			
velocity	*V1*	*V2*	*V3*	*V4*	*V1*	*V2*	*V3*	*V4*
tracer (deg/s)	45.00	89.86	180.05	270.76	44.92	90.07	180.36	270.54
target (deg/s)	45.00	90.00	180.00	270.00	45.00	90.00	180.00	270.00
tracer/target (%)	99.99	99.85	100.03	100.28	99.83	100.08	100.20	100.20
std (deg/s)	0.13	0.25	0.72	1.19	0.21	0.49	0.97	3.20
std/tracer (%)	0.29	0.28	0.40	0.44	0.47	0.54	0.54	1.18

## Discussion

This research aims to examine motor control strategies based on the reliability of visual information on the frontal and sagittal planes for circular tracking movements. The results demonstrated that the subjects performed the tracking movements to reduce position errors between the target and the tracer when the reliability of visual information was superior. This was shown by a minor ΔR during performances on the frontal plane and the velocity of the target is slow. In contrast, the reduced reliability of visual information led to periodic motor performance based on a particular location being the purpose of motor control. This can be confirmed by the value of ΔR, which depends on the quadrant of the trajectory, and by the frequency analysis of Δω.

The following topics are discussed in the following sections: (1) identification of optimal feedback control for circular tracking movement, (2) relationship between visual information and rhythmic movement, and (3) future work.

### Identification of optimal feedback control for circular tracking movement

A plane is 2D space consisting of two orthogonal coordinate axes, and it is essential to identify the relevant axes in order to accurately comprehend the position of an object on a certain plane. For instance, the x- and y-axes are dominant factors in movements on the frontal plane. Both axes are parallel to the subjects, and any positional changes of the target and the tracer can be spotted on the x- and y-axes. Therefore, visual information obtained from the frontal plane is highly accurate [16–18]. However, on the sagittal plane, the y- and z-axes are dominant components. Positional changes on the y-axis can be easily detected because the axis is parallel to the subjects; however, for the z-axis, it is difficult to observe its positional changes, because it contains depth information in the orthogonal direction from the subject’s view. The axis can only be estimated by varying the sizes of the target and the tracer. This indicates that obtaining accurate visual feedback is relatively challenging on the sagittal plane [16–18].

Motor uncertainty increases when the accuracy of sensory information decreases or is hindered by the presence of noise in human movements [28]. According to the study by Yuille and Kersten, visuo-motor uncertainty is derived from the ambiguity of projecting 3D information on a 2D retina [29]. Moreover, our previous studies illustrated that the accuracy of visual information was reduced when the target and the tracer became distant from the subjects [16–18]. These discoveries indicate that insufficiency of visual information increased the motor uncertainty and consequently allowed larger *ΔR* between the target and the tracer on the sagittal plane than on the frontal plane. In fact, *ΔR* increased when the target velocity increased, irrespective of the planes. Meaning, the growth in ΔR, observed with the increased target velocity, could be explained by an increase in motor uncertainty due to ambiguity related to the sensory information. Potential causes of motor uncertainty may also be muscular fatigue or the fact that different muscles are utilized in different planar movements. The reasons will be investigated in detail in future studies.

The results of the circular tracking movements can be interpreted as multisensory integration based on Bayesian decision theory [30–32]. The most dominant sensory feedbacks in the given experimental setup were visual and proprioceptive information. Neural weights of the feedback are determined by their sensory reliabilities and transfer information with respect to on motor performance [30–32]. Van Beers and Wolpert discovered that the weight of the visual information is larger on the frontal plane, whereas the proprioceptive information retains more weight in accordance with depth on the sagittal plane [15]. However, this study analyzed the reaching movement, and the purpose location of the movement was one point. On the other hand, in the circular tracking movement performed in our study, the purpose location of movement is the number of points on the trajectory. Through this, we inferred that there would be a difference between the sensory information acquired at each target position in the tracking movement. In particular, on the sagittal plane, the reliability of visual information appears to vary depending on the depth difference. If this is applied to the Bayesian decision theory, it can be inferred that the weights of visual information and proprioception information change during the tracking exercise. To confirm this, we divided the trajectory into four quadrants and checked the value of *ΔR*.

The characteristics of motor performance in each plane were analyzed to obtain a planar comparison of the control strategies. By dividing one orbit of the target’s trajectory into four quadrants, the changes in *ΔR* throughout all quadrants of the frontal plane could be considered trivial (Figs 3 and 9A). The weight of the visual information did not change because the distance between the subject and the target on the z-axis remains identical for all quadrants [15]. On the sagittal plane, the second quadrant had the smallest value of *ΔR*, while the fourth quadrant had the greatest *ΔR* (Figs 4 and 9B). The second quadrant can be considered as the section where the visual information contains the most sensory weight because the quadrant is the closest to the subject’s eye compared to other quadrants. In contrast, the fourth quadrant is located at the farthest distance from the eye, and is examined to cause a decrease in the weight of the visual information and the rise of the proprioceptive weight [15]. In general, proprioceptive information provides less accurate feedback than visual information [32, 33]. This infers that the accuracy of the tracking movement is increased in the second quadrant and reduced in the fourth quadrant. However, when a change in direction occurs from downward movement to upward movement affected by the direction of gravity, a change in contraction of the muscles may occur. This may be the cause of the difference in *ΔR* depending on the quadrant in which the target and tracer are located. Since measurements have not been made in the present study, it is necessary to confirm this in future studies.

Inoue and Sakaguchi investigated target tracking movements by manipulating a vertically movable slider [12]. The target was repeatedly moved in a sinusoidal waveform on a 1D line by adjusting its velocity and direction, and the subject was to control the slider to move the cursor displayed on a screen. The experiment was designed to be performed under five different conditions: target visible at full trajectory, target removal at the top turning point, at the center with upward, at the bottom turning point, and at the center with downward. In summary, the condition of the visual feedback during the tracking movement was periodically modified. From the results of Inoue and Sakaguchi’s study, it can be inferred that the area with the visible target allowed movements with high weight on the visual-feedback control, whereas the subjects executed movements with high weight on feedforward control within the target-removed area.

The subjects could only detect y-axis displacements, and hardly noticed z-axis displacements of both the target and the tracer visually when they conducted the tracking movement on the sagittal plane. Furthermore, the visual condition of the circular tracking movement on the sagittal plane periodically changed with the difference in depth information. This would make the main use of information only in one axis, and would lead to repeated adjustment of visual information’s weight, which indicates some similarity to visual conditions from Inoue and Sakaguchi’s experiment. According to their study, the position error of the tracer on the 1D trajectory tended to be minimized in a particular section, although the location of the section differed depending on the provided visual condition [12]. This indicates that the brain does not continuously monitor ongoing motor performance but rather executes movements with the aim of having high accuracy on certain points, implying that the visual condition is a dominant factor that influences movements [12].

The result of the circular tracking movement in the sagittal plane retained the least *ΔR* in the second quadrant, irrespective of the target velocity (Fig 9). Similar to Yasuyuki and Yutaka’s study, our results of the tracking movement were examined to minimize *ΔR* at certain points. This could be considered similar to the experimental results obtained by the periodic weight change of similar visual information. This inference may be controversial because particular arm kinematics may be the cause of the minimization of *ΔR* at certain points. Aspects of the arm kinematics could be different if the subjects conduct the tracking movement in the opposite direction, so differences in motor performance in counterclockwise circular tracking movements will be examined in future studies.

The OFC is a control method that integrates feedforward-based optimization and feedback control to ensure the accuracy of a movement goal [34]. In other words, humans actively utilize feedback for an area related to the given movement goal, and feedforward control is performed to reduce the cost in a less relevant area [34]. Van Beers and Wolpert observed that the distance of target decreases, the weight of visual information is relatively high, and the subject performs active feedback control [15]. On the other hand, the weight of the proprioceptive information is high and feed-forward control is performed further with increment of target distances [15]. In this study, it can also be inferred that the subjects performed active visual feedback control in the second quadrant where the distance to the target is close, compared with the results on the sagittal plane, minimizing *ΔR* in the second quadrant could be interpreted as a goal of the tracking movement. Other quadrants can be considered as areas where feedforward control is more dominant when moving the tracer along the predicted trajectory than actively utilizing feedback. In these areas, *ΔR* is believed to increase owing to the absence of active feedback for optimized movements. Thus, motor control is optimized by actively applying feedback control to aspects directly related to accomplishing the movement goal, and by minimizing interference for irrelevant tasks [34–38].

The circular tracking movement on the sagittal plane was analyzed in order to determine limitations in position feedback control because of the weight change of the visual information. As a result, the circular tracking movement appeared to minimize the *ΔR* through feedback control in the second quadrant, where the weight of the visual information is the largest. However, in the other quadrants, the OFC was applied to the tracking movement by optimally adopting feedforward control. On the frontal plane, the *ΔR* throughout all four quadrants was trivial, and the deviation of *ΔR* was approximately three times greater on the sagittal plane than on the frontal plane. This clearly indicates that the tracking movement on the frontal plane utilizes feedback control as a primary control mechanism for all areas because the frontal plane provides high accuracy of the visual information on the target through the entire trajectory.

### Relationship between visual information and rhythmic movement

If the target is moving at a relatively slow velocity (*V1* or *V2*), the *ω* value of the tracer is less than that of the target at all points in time, regardless of the planes. In contrast, *Δω* increases if the target moves at a relatively high velocity (*V3* or *V4*). The extent of the increase was greater on the sagittal plane than on the frontal plane, and all increases showed a rhythmic change (Figs 5–8).

The prediction control is suppressed under the conditions of an error correction function, which can be operated during a slow or tracking motion with sufficient visual information within the entire trajectory [26]. However, if the error correction function does not work because of insufficient information from the surrounding environment, the prediction function is more likely to operate, and while the prediction function is performed, the rhythm acquired from the surrounding environment is used for prediction control. In other words, the degree of involvement of the predictive control depends on the reliability of the visual information, and it can be confirmed based on the rhythmic degree of *ω* [26].

An FFT analysis was conducted on *ω* to obtain a more quantitative comparison of the rhythmic degree. The amplitude spectrum was compared within the low-frequency range of 0–5 Hz at each velocity. Based on the result, the amplitude spectrum is larger than that of the frontal plane when the circular tracking movement is applied on the sagittal plane.

In addition, the amplitude spectrum was larger during the fast motion than during the slow motion of the target. This is similar to the frequency analysis of visible and invisible movements [26]. Hence, if the visual information is uncertain during a circular tracking movement, the rhythmic tendency increases.

The analysis of *ω* can be considered to be related to the OFC. When the target moves at *V3* and *V4*, the *ω* values of the tracer and target indicate errors according to each quadrant position. The average values of *ω* were almost identical between the target and tracer during one complete cycle (Table 4). Therefore, the rapid tracking movement of the sagittal plane minimizes the *ΔR* of a specific location (quadrant 2). We confirmed the tendency to control the rotation period of the tracer to match the rotation period of the target based on quadrant 2. Even along the frontal plane, a similar tendency was observed when the target moved at *V3* and *V4*. In previous studies by Yoshikatsu and Yurie, there was a control tendency that subject matched the periodicity of the target and the tracer based on a specific area when performing a periodic tracking movement [26]. Similarly, the goal of the tracking movement in this study can be interpreted as matching the periodicity of the target and the tracer. The specific area in this experiment can be deduced as the second quadrant where *ΔR* becomes the smallest, as discussed previously. Therefore, the goal of the circular tracking movement is to match the periodicity of the target and the tracer, and the area as the reference can be considered as the second quadrant in OFC theory. The second quadrant is a position related to the goal of the movement, and the other quadrants can be viewed as less related areas.

### Future work: Confirmation of learning effect, noise impact reduction, and application to rehabilitation

In this study, the subjects were asked to exercise within the first environment they experienced. In this case, we found that the weight of the visual information was larger than the weight of the proprioceptive information. In the future, the subjects will be sufficiently trained and then asked to conduct the same exercise. It is expected that the weight of the proprioceptive information will increase and that the weight of the visual information will decrease.

During this experiment, the controller was held in the subject’s hand, and the target was traced with the end point of the controller. This required control of the position and state of the controller, which means that precise control is difficult to achieve and that a time delay may occur. This situation may have caused noise in the experimental results. If the subject traces the target by hand without holding the controller, the subject will be able to reduce the effects of noise before the pre-processing of the analysis results.

There have been various cases involving controlling visual feedback and applying it to rehabilitation. However, the effects of rehabilitation under various visual feedback situations were not analyzed; therefore, quantitative evaluation was limited [39–42]. We were able to compare the differences in the control strategy according to the accuracy of the visual information. If the same study is conducted on hemiplegic patients, it will be possible to identify changes in the patient’s control strategy after a brain injury. In addition, the parameters measured in normal persons and those measured in patients with an ailment can be compared and applied to a quantitative assessment of the patient’s condition, and may be applicable as a standard for establishing an appropriate visual feedback environment when conducting rehabilitation treatment.

## Conclusions

In this study, we analyzed the control strategy of a circular tracking movement in 3D space according to the accuracy of the visual information. The value of *ΔR* was shown to be two to three times greater in quadrants 1 and 4 than in quadrants 2 and 3 near the subject. We also found that the value of *ΔR* increased as the velocity of the target increased. This indicates that the spatial information of the visuo-motor control is significantly influenced by the accuracy of the visual information owing to the depth and target velocity. When *ω* was analyzed within the frequency domain, the periodicity was shown to be approximately 1.7 to 2 times stronger on the sagittal plane than on the frontal plane, and as the velocity of the target increased, the periodicity also increased. This means that the periodicity of movement is also affected by the accuracy of the visual information based on the depth and target velocity. However, the average *ω* value of the tracer was similar to that of the target (i.e., within 0.6%) during one trial. In other words, the *ω* values of the tracer and the target are not the same at all positions, although the same time is required to perform a single trial.

From the characteristics of the aforementioned two parameters (R, ω), it can be inferred that the subject performs a periodic circular tracking motion in the sagittal plane and matching the periodicity in the second quadrant for the purpose of exercise.

Based on the characteristics of the two aforementioned parameters (R, ω), it can be inferred that the subject performs a periodic circular tracking movement on the sagittal plane and matching the periodicity in the second quadrant for the goal of tracking. If this is applied to OFC control, the second quadrant with high reliability of visual information can be thought as an area that is highly related to the goal of tracking, and the other quadrants can be thought as areas those are not related to the goal of tracking. Accordingly, we can infer that the subjects have a high weight of the visual feedback control in the second quadrant, and that the movement with a high weight of the feed forward control performs in the other quadrant where the reliability of visual information is low. However, since the movement characteristics of the arm structure may be reflected, further research is required.

## Supporting information

S1 Table Summary of the statistical analysis of *ΔR* in the frontal plane. (DOCX)

S2 Table Summary of the statistical analysis of *ΔR* in the sagittal plane. (DOCX)
