# Supplementary material for: Analysis of motor control strategy for frontal and sagittal planes of circular tracking movements using visual feedback noise from velocity change and depth information
Source: PLoS One. 2020 Nov 11;15(11):e0241138. doi: 10.1371/journal.pone.0241138 (PMC7657550; doi:10.1371/journal.pone.0241138)
Supplement: S1 Table — (DOCX) [file pone.0241138.s001.docx]

**S1 Table.** **Summary of statistical analysis of** $\boldsymbol{\Delta}\boldsymbol{R}$ **on the frontal plane.**

| **Item** | **Variable** | **Test** | **Statistic** | **Confidence** |
| --- | --- | --- | --- | --- |
| A | ΔR under the conditions of each quadrant at each target speed | Two-way repeated measures  ANOVA | Frequency (target velocity):  Mauchly's Test χ2(5) = 27.819,  p = 0, ε = 0.592;  F (1.776,44.398) = 48.675;  Quadrant:  Mauchly's Test χ2(5) = 13.817, p = 0.017, ε = 0.722;  F (2.166,54.143) = 9.658;  Interaction:  Mauchly's Test χ2(44) = 81.33, p = 0.001, ε = 0.579;  F (5.213,130.335) = 1.752 | Frequency (target velocity): p = 0, partial η2 = 0.661,  power = 1,  corrected by Greenhouse-Geisser;    Quadrant: p = 0, partial η2 = 0.279,  power = 0.982,  corrected by Greenhouse-Geisser;  Interaction: p = 0.124, partial η2 = 0.065, power = 0.601,  corrected by Greenhous-Geisser |
| B | ΔR under the conditions of Q1:Q2, Q1:Q3, Q1:Q4, Q2:Q3, Q2:Q4, Q3:Q4 in V1 | Bonferroni-corrected pairwise comparisons | Q1: Q2: t (25) = 2.423;  Q1: Q3: t (25) = 0.127;  Q1: Q4: t (25) = 3.469;  Q2: Q3: t (25) = 2.837;  Q2: Q4: t (25) = 3.894;  Q3: Q4: t (25) = 3.322 | Q1: Q2; p = 0.138, Cohen’s d = 0.475;  Q1: Q3: p = 1.000, Cohen’s d = 0.025;  Q1: Q4: p = 0.011, Cohen’s d = 0.682;  Q2: Q3: p = 0.053, Cohen’s d = 0.556;  Q2: Q4: p = 0.004, Cohen’s d = 0.764;  Q3: Q4: p = 0.017, Cohen’s d = 0.652; |
| C | ΔR under the conditions of Q1:Q2, Q1:Q3, Q1:Q4, Q2:Q3, Q2:Q4, Q3:Q4 in V2 | Bonferroni-corrected pairwise comparisons | Q1: Q2: t (25) = 0.660;  Q1: Q3: t (25) = 1.548;  Q1: Q4: t (25) = 2.236;  Q2: Q3: t (25) = 2.844;  Q2: Q4: t (25) = 2.089;  Q3: Q4: t (25) = 0.901 | Q1: Q2; p = 1.000, Cohen’s d = 0.129;  Q1: Q3: p = 0.805, Cohen’s d = 0.304;  Q1: Q4: p = 0.207, Cohen’s d = 0.438;  Q2: Q3: p = 0.053, Cohen’s d = 0.558;  Q2: Q4: p = 0.282, Cohen’s d = 0.410;  Q3: Q4: p = 1.000, Cohen’s d = 0.177 |
| D | ΔR under the conditions of Q1:Q2, Q1:Q3, Q1:Q4, Q2:Q3, Q2:Q4, Q3:Q4 in V3 | Bonferroni-corrected pairwise comparisons | Q1: Q2: t (25) = 1.342;  Q1: Q3: t (25) = 0.529;  Q1: Q4: t (25) = 1.251;  Q2: Q3: t (25) = 0.932;  Q2: Q4: t (25) = 2.051;  Q3: Q4: t (25) = 1.518 | Q1: Q2; p = 1.000, Cohen’s d = 0.263;  Q1: Q3: p = 1.000, Cohen’s d = 0.104;  Q1: Q4: p = 1.000, Cohen’s d = 0.245;  Q2: Q3: p = 1.000, Cohen’s d = 0.183;  Q2: Q4: p = 0.305, Cohen’s d = 0.402;  Q3: Q4: p = 0.850, Cohen’s d = 0.298; |
| E | ΔR under the conditions of Q1:Q2, Q1:Q3, Q1:Q4, Q2:Q3, Q2:Q4, Q3:Q4 in V4 | Bonferroni-corrected pairwise comparisons | Q1: Q2: t (25) = 1.861;  Q1: Q3: t (25) = 0.891;  Q1: Q4: t (25) = 1.563;  Q2: Q3: t (25) = 2.799;  Q2: Q4: t (25) = 3.581;  Q3: Q4: t (25) = 1.435 | Q1: Q2; p = 0.447, Cohen’s d = 0.365;  Q1: Q3: p = 1.000, Cohen’s d = 0.175;  Q1: Q4: p = 0.784, Cohen’s d = 0.307;  Q2: Q3: p = 0.058, Cohen’s d = 0.549;  Q2: Q4: p = 0.009, Cohen’s d = 0.702;  Q3: Q4: p = 0.982, Cohen’s d = 0.281; |
