# Supplementary material for: Analysis of motor control strategy for frontal and sagittal planes of circular tracking movements using visual feedback noise from velocity change and depth information
Source: PLoS One. 2020 Nov 11;15(11):e0241138. doi: 10.1371/journal.pone.0241138 (PMC7657550; doi:10.1371/journal.pone.0241138)
Supplement: S2 Table — (DOCX) [file pone.0241138.s002.docx]

**S2 Table. Summary of statistical analysis of** $\boldsymbol{\Delta}\boldsymbol{R}$ **on the sagittal plane.**

| **Item** | **Variable** | **Test** | **Statistic** | **Confidence** |
| --- | --- | --- | --- | --- |
| A | ΔR under the conditions of each quadrant at each target speed | Two-way repeated measures  ANOVA | Frequency (target velocity):  Mauchly's Test χ2(5) = 3.615,  p = 0.606, ε = 1;  F (3,75) = 34.941;  Quadrant:  Mauchly's Test χ2(5) = 30.054, p = 0, ε = 0.697;  F (2.092,52.288) = 56.260;  Interaction:  Mauchly's Testχ χ2(44) = 130.399, p = 0, ε = 0.549;  F (4.940,123.488) = 0.362 | Frequency (target velocity): p = 0, partial η2 = 0.583,  power = 1,  corrected by Huynh-Feldt    Quadrant: p = 0, partial η2 = 0.692,  power = 1,  corrected by Greenhouse-Geisser;  Interaction: p = 0.872, partial η2 = 0.014, power = 0.140,  corrected by Greenhous-Geisser |
| B | ΔR under the conditions of Q1:Q2, Q1:Q3, Q1:Q4, Q2:Q3, Q2:Q4, Q3:Q4 in V1 | Bonferroni-corrected pairwise comparisons | Q1: Q2: t (25) = 2.378;  Q1: Q3: t (25) = 2.405;  Q1: Q4: t (25) = 5.780;  Q2: Q3: t (25) = 0.416;  Q2: Q4: t (25) = 6.246;  Q3: Q4: t (25) = 6.445 | Q1: Q2; p = 0.138, Cohen’s d = 0.466;  Q1: Q3: p = 1.000, Cohen’s d = 0.472;  Q1: Q4: p = 0.011, Cohen’s d = 1.134;  Q2: Q3: p = 0.053, Cohen’s d = 0.082;  Q2: Q4: p = 0.004, Cohen’s d = 1.225;  Q3: Q4: p = 0.017, Cohen’s d = 1.264 |
| C | ΔR under the conditions of Q1:Q2, Q1:Q3, Q1:Q4, Q2:Q3, Q2:Q4, Q3:Q4 in V2 | Bonferroni-corrected pairwise comparisons | Q1: Q2: t (25) = 4.120;  Q1: Q3: t (25) = 2.308;  Q1: Q4: t (25) = 4.889;  Q2: Q3: t (25) = 1.744;  Q2: Q4: t (25) = 5.545;  Q3: Q4: t (25) = 5.139 | Q1: Q2; p = 1.000, Cohen’s d = 0.808;  Q1: Q3: p = 0.805, Cohen’s d = 0.453;  Q1: Q4: p = 0.207, Cohen’s d = 0.959;  Q2: Q3: p = 0.053, Cohen’s d = 0.342;  Q2: Q4: p = 0.282, Cohen’s d = 1.087;  Q3: Q4: p = 1.000, Cohen’s d = 1.008 |
| D | ΔR under the conditions of Q1:Q2, Q1:Q3, Q1:Q4, Q2:Q3, Q2:Q4, Q3:Q4 in V3 | Bonferroni-corrected pairwise comparisons | Q1: Q2: t (25) = 2.304;  Q1: Q3: t (25) = 0.651;  Q1: Q4: t (25) = 4.904;  Q2: Q3: t (25) = 1.979;  Q2: Q4: t (25) = 6.816;  Q3: Q4: t (25) = 5.277 | Q1: Q2; p = 1.000, Cohen’s d = 0.452;  Q1: Q3: p = 1.000, Cohen’s d = 0.128;  Q1: Q4: p = 1.000, Cohen’s d =0.962;  Q2: Q3: p = 1.000, Cohen’s d = 0.388;  Q2: Q4: p = 0.305, Cohen’s d = 1.337;  Q3: Q4: p = 0.850, Cohen’s d = 1.035 |
| E | ΔR under the conditions of Q1:Q2, Q1:Q3, Q1:Q4, Q2:Q3, Q2:Q4, Q3:Q4 in V4 | Bonferroni-corrected pairwise comparisons | Q1: Q2: t (25) = 2.345;  Q1: Q3: t (25) = 1.224;  Q1: Q4: t (25) = 3.212;  Q2: Q3: t (25) = 1.281;  Q2: Q4: t (25) = 6.503;  Q3: Q4: t (25) = 7.052 | Q1: Q2; p = 0.447, Cohen’s d = 0.460;  Q1: Q3: p = 1.000, Cohen’s d = 0.240;  Q1: Q4: p = 0.784, Cohen’s d = 0.630;  Q2: Q3: p = 0.058, Cohen’s d = 0.251;  Q2: Q4: p = 0.009, Cohen’s d = 1.275;  Q3: Q4: p = 0.982, Cohen’s d = 1.383 |
